# The impact of test loads on the accuracy of 1RM prediction using the load-velocity relationship

**DOI:** 10.1186/s13102-018-0099-z

**Published:** 2018-05-29

**Authors:** Mark G. L. Sayers, Michel Schlaeppi, Marina Hitz, Silvio Lorenzetti

**Affiliations:** 1 School of Health and Sport Sciences, University of the Sunshine Coast, Maroochydore DC, QLD 4558 Australia; 2 Institute for Biomechanics, ETH Zürich, Zürich, Switzerland; 3 Swiss Federal Institute of Sport, Magglingen, Switzerland

**Keywords:** Strength assessment, Dynamic strength, Predictive models, Bench press throws

## Abstract

**Background:**

Numerous methods have been proposed that use submaximal loads to predict one repetition maximum (1RM). One common method applies standard linear regression equations to load and average vertical lifting velocity (V_mean_) data developed during squat jumps or three bench press throw (BP-T). The main aim of this project was to determine which combination of three submaximal loads during BP-T result in the most accurate prediction of 1RM Smith Machine bench press strength in healthy individuals.

**Methods:**

In this study combinations of three BP-T loads were used to predict 1RM Smith Machine bench press strength. Additionally, we examined whether regression models developed using peak vertical bar velocity (V_peak_), rather than V_mean_, provide the most accurate prediction of Smith Machine bench press 1RM. 1RM Smith Machine bench press strength was measured directly in 12 healthy regular weight trainers (body mass = 80.8 ± 5.7 kg). Two to three days later a linear position transducer attached to the collars on a Smith Machine was used to record V_mean_ and V_peak_ during BP-T between 30 and 70% of 1RM (10% increments).

**Results:**

Repeated measures analysis of variance testing showed that the mean values for slope and ordinate intercept for the regression models at each of the load ranges differed significantly depending on whether V_mean_ or V_peak_ were used in the prediction models (*P* < 0.001). Conversely, the abscissa intercept did not differ significantly between either measure of vertical bar velocity at each load range. The key finding in this study was that 1RM Smith Machine bench press strength can be determined with high relative accuracy by examining V_mean_ and V_peak_ during BP-T over three loads, with the most precise models using V_peak_ during loads representing 30, 40 and 50% of 1RM (*R*^*2*^ = 0.96, *SSE* = 4.2 kg).

**Conclusions:**

These preliminary findings indicate that exercise programmers working with normal healthy populations can accurately predict Smith Machine 1RM bench press strength using relatively light load Smith Machine BP-T testing, avoiding the need to expose their clients to potentially injurious loads.

## Background

The quantification of the maximum load that can be lifted through a full range of motion, or one repetition maximum (1RM), is fundamental to the design of resistance training programs [1]. Typically, 1RM is either measured directly or calculated indirectly using predictive models. The direct determination of 1RM suffers from a number of pragmatic issues as it is not only time consuming, but the outcome is effected by factors such as athlete experience, technique, fatigue and motivation [2]. Traditional 1RM testing is considered to be safe when it is conducted in appropriate settings under the supervision of qualified practitioners [3, 4]. Regardless, this 1RM exposes athletes to large musculoskeletal forces, and there is some evidence that 1RM testing can be potentially injurious [5, 6] and may also be impractical with novices and/or in clinical settings [7].

Indirect methods for 1RM quantification tend to follow two different protocols, both of which rely on the use of linear regression modelling. The most common indirect protocols involve lifting submaximal loads to failure [7–9], a procedure that is relatively common in trained athletes [10] and in agreement with the ACSM guidelines of 8–12 repetition that is often used in a clinical setting, but rare in everyday activities. Although relatively easy to administer, the accuracy of these ‘lift to failure’ models is also influenced by elements such as age, training experience, motivation and lifting tempo [7–9, 11]. The prediction of 1RM using these methods appears to be more accurate when heavier loads are used [12–14], with the optimal number of repetitions for these prediction models being less than 10 [8]. Accordingly, these protocols potentially suffer from the same limitations associated with 1RM testing, with the need to lift high relative loads whilst fatigued. Additionally, these lift to failure protocols are also likely to generate post exercise muscle soreness in novices [15], potentially dissuading them from future exercise participation.

Alternative indirect methods rely on the load-velocity [6, 16] or force-velocity [10, 11, 17–19] relationships and linear or quasi linear models to predict 1RM from a series of maximal effort lifts with submaximal loads. These protocols use either isoinertial sensors or linear position transducers that are attached to the collars or bar of training devices like Smith Machines to record force, average and/or peak vertical lifting velocity data from the concentric phase of movements like jump squats or bench press throws (BP-T). Although these movements are more common in high performance training programs, the use of a Smith Machine and appropriately trained “*Spotters*” means that these exercises can be completed safely with novice participants (NB: some Smith Machines contain a pneumatic brake which prevents the bar from descending rapidly – hence improving exercise safety). Although 1RM data recorded on Smith Machines are typically 10% higher than those recorded using free weights, there are no significant differences between predicted 1RM values when using these devices [20]. These protocols also have the advantage of being relatively quick to perform as they involve loads between 30 and 80% of 1RM [11, 16, 21] being lifted as rapidly as possible for only 2–4 repetitions. Accordingly, the overall loading in these protocols is less than ‘lift to failure’ protocols and so the risk of injury may be decreased, particularly when applied to relatively untrained populations [16].

Arguably, the simplest of the load-velocity models [16] applies standard linear regression equations to load and mean vertical propulsive lifting velocity (V_mean_) data from three different loads to develop slope, abscissa (Load0) and ordinate (V_mean_0) intercept data. Importantly, variables such as V_mean_ or peak vertical velocity (V_peak_) can be measured using relatively inexpensive technology that, due to large reductions in pricing, is becoming increasing accessible to strength coaches. Researchers report high correlations using this methods between Load0 and 1RM bench press (*r* = 0.98, *n* = 112, *SEE* = 4 kg [7%]), although the strength of this relationship is no doubt influenced by the large range in relative loads assessed (30 to 95% of 1RM) [16, 22]. Additionally, participants in the study by Jidovtseff and co-workers [16] were required to always hold the bar (i.e. prevented from performing a BP-T), which will have a marked effect on V_mean_ due to the deceleration of the bar near the end of the lift [23]. Nevertheless, some questions remain as to whether V_mean_ or V_peak_ provides superior predictive measures. Recently, Gracia-Ramos and co-workers [24] report that V_mean_ during Smith Machine bench press is a superior predictor of 1RM when compared to V_peak_. However, these findings appear to be specific to the testing protocols as these researchers highlight in another study that V_peak_ during bench press throws is the superior predictor of 1RM [25]. Regardless, V_mean_ and V_peak_ appear to be greater predictors of optimal load for power training than traditional methods that advocate percentages of 1RM [26].

Nevertheless, the question remains as to the efficacy of the procedures proposed by Jidovtseff and co-workers [16], particularly when testing novice or inexperienced weight trainers for which higher lifting loads may be contraindicated. Therefore, it is important to determine whether such high relative loads are required during these submaximal test protocols (i.e. up to 95% of 1RM) and which combination of relative loads result in the most accurate predictive model of 1RM bench press strength. Accordingly, the purpose of this study was to use the prediction model developed by Jidovtseff and coworkers [16] to determine which combination of three submaximal loads during BP-T result in the most accurate prediction of 1RM Smith Machine bench press strength in healthy individuals. We also examined whether the ability to release the bar during the BP-T changes the nature of the prediction model. Additionally, we examined whether V_mean_ or V_peak_ provides a better prediction of Smith Machine bench press 1RM strength in these participants.

## Methods

### Experimental approach to the problem

To determine which combination of three loads during BP-T results in the most accurate prediction of 1RM bench press strength we tested 12 healthy, regular weight trainers on two separate occasions. On the first occasion 1RM bench press strength was recorded using standard procedures and recorded to the nearest 1 kg [27]. During the second data collection (2–3 days after the first testing session) participants performed three repetitions of BP-T at loads representing 30, 40, 50, 60 and 70% of their 1RM. We then processed these load-velocity BP-T data using the techniques proposed by Jidovtseff and coworkers [16] to determine which three load range (30–50% of 1RM, 40–60% of 1RM, 50–70% of 1RM) resulted in the most accurate prediction of 1RM bench press strength. We also examined whether BP-T V_mean_ or V_peak_ provides a more accurate prediction of bench press 1RM by comparing each of the models developed using these variables.

### Participants

Participants for this study (*n* = 12) were all recreational weight trainers who had been weight training at least twice a week for a minimum of 1 year (body mass (BM) = 80.8 ± 5.7 kg, 1RM 84 ± 18 kg, relative 1RM =1.04 BM [i.e. relative load is represented as a function of BM]). None of the participants were involved in heavy load strength training. Participants were informed of the experimental procedures and risks and provided their written informed consent prior to attending several familiarisation sessions. This research was approved by the institutional Human Research Ethics Committee (No. 2012-N-10).

### Procedures

All bench press and BP-T data were collected on a standard Smith Machine. This machine was modified with a custom made magnetic braking system as a safety mechanism. Once the bar was released this safety mechanism prevented it from falling back on the participant [2]. The bar handle was attached permanently to this braking system, resulting in a total weight of 23 kg. To record the vertical position of the bar a linear position transducer (LPT) (WS17KT, ASM, Moosinning, Germany) was installed on the Smith Machine’s pneumatic brake, with data subsequently sampled at 1000 Hz, A/D converted and stored on a computer, Subsequent data analysis of the LPT measurement were performed in MATLAB, with velocity data developed from the raw LPT outputs using the first central difference method.

The BP-T testing was conducted in accordance with well-established protocols [23, 28, 29] at loads representing 30, 40, 50, 60 and 70% relative to 1RM. The execution order was determined randomly using Microsoft Excel in order to avoid possible order effects during the testing session. In order to minimise the effects of fatigue there were 2–4 min between repetitions with three repetitions completed at each load. The eccentric phase was at a self-chosen speed, with the participants required to wait for the start signal before commencing the concentric motion [30]. There was approximately at 2 s pause between the eccentric and concentric phases.

**Fig. 1:**
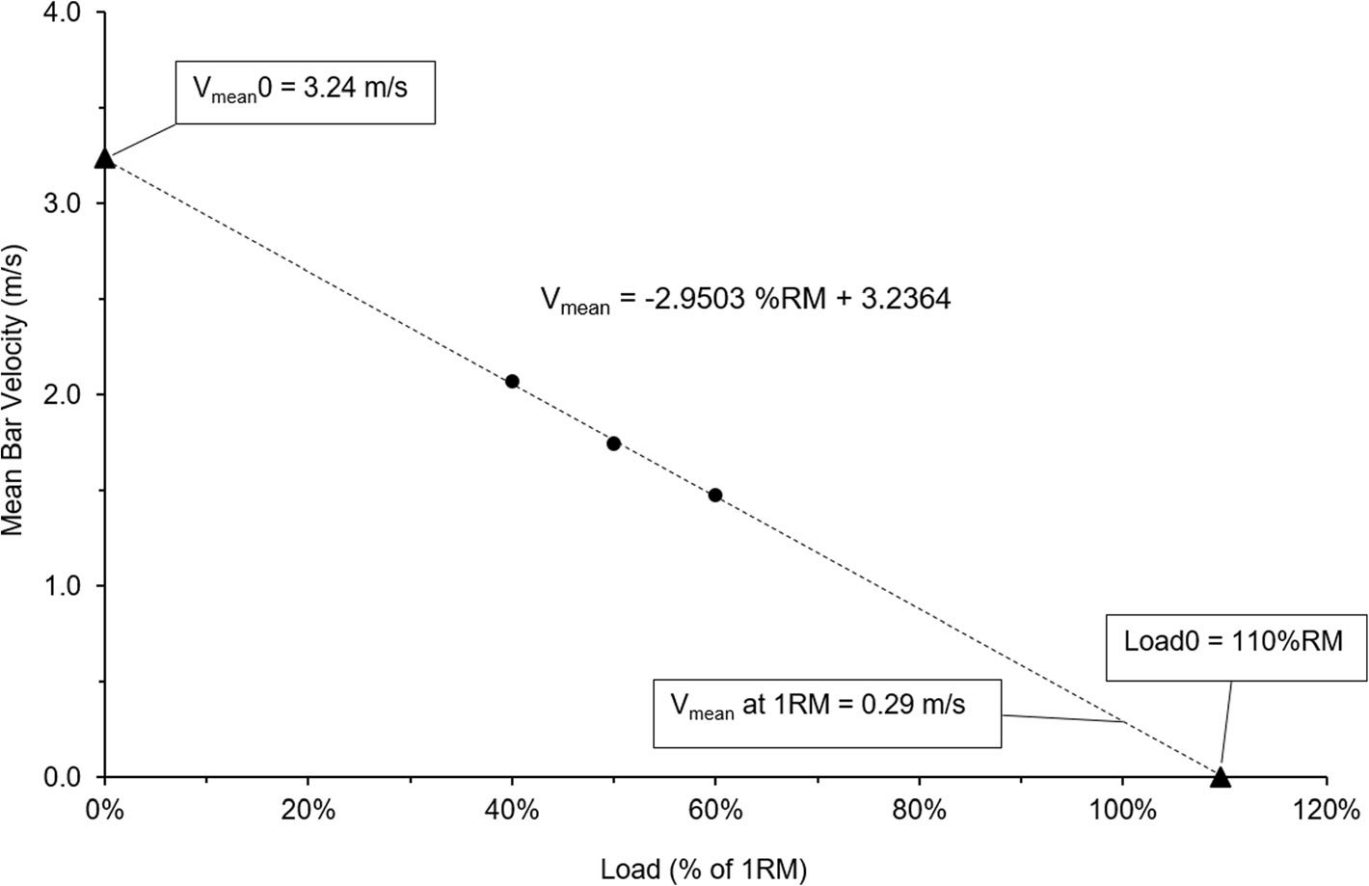
Sample data from one subject, three loads (solid circles) processed using standard load-velocity techniques [16]. Graph includes the regression line and the calculated peak mean vertical velocity (V_mean_0), theoretical load at 0 m/s (Load0) and average vertical lifting velocity (V_mean_) at 1RM

The V_mean_, and V_peak_ and maximum bar acceleration were calculated from the first and second differentials of the linear transducer data. These data were then used to develop a linear regression model for the prediction of 1RM [16] (Fig. 1). We subsequently developed slope, Load0 and V_mean_0 data for each of these regressions models over each of the load ranges (i.e. 30–50% 1RM, 40–60% 1RM and 50–70% 1RM).

### Statistical analyses

The influence of load on the various bar kinematic variables were determined via a series of repeated measures analysis of variance (ANOVA) tests. Post-hoc analyses were undertaken using paired t-Test with Bonferroni corrections. Shapiro-Wilk and Mauchly’s test of sphericity were applied during all ANOVA testing. Where data violated the sphericity assumption Greenhouse-Geisser corrections were applied. The relative magnitude of differences were quantified using standard Cohen’s Effect Size (*ES*) analyses, with the following descriptors used to define the relative magnitude of the ES: < 0.2 = *trivial*, 0.2–0.6 = *small*, 0.6–1.2 *= medium/moderate*, 1.2–2.0 = *large*, and > 2.0 = *very large* [31]. The predictive accuracy of the model developed by Jidovtseff and coworkers [16] was assessed using the three lightest loads (30–50% 1RM), the three middle loads (40–60% 1RM) and the three heaviest loads (50–70% 1RM), with these data then compared with the measured 1RM values. Bland-Altman plots were used to assess whether there were any levels of bias in any of the models, with simple t-tests used to assess for differences between the actual and predicted values. The coefficient of variance (CV%) and the intra class correlations (ICC, 3,1) for the predicted versus the measured 1RM were also calculated. Statistical analysis were performed using the statistics package SPSS for Windows (version 20), with a confidence level of 95%. All data are presented at means ±1 standard deviation (SD) unless stated otherwise.

## Results

**Fig. 2:**
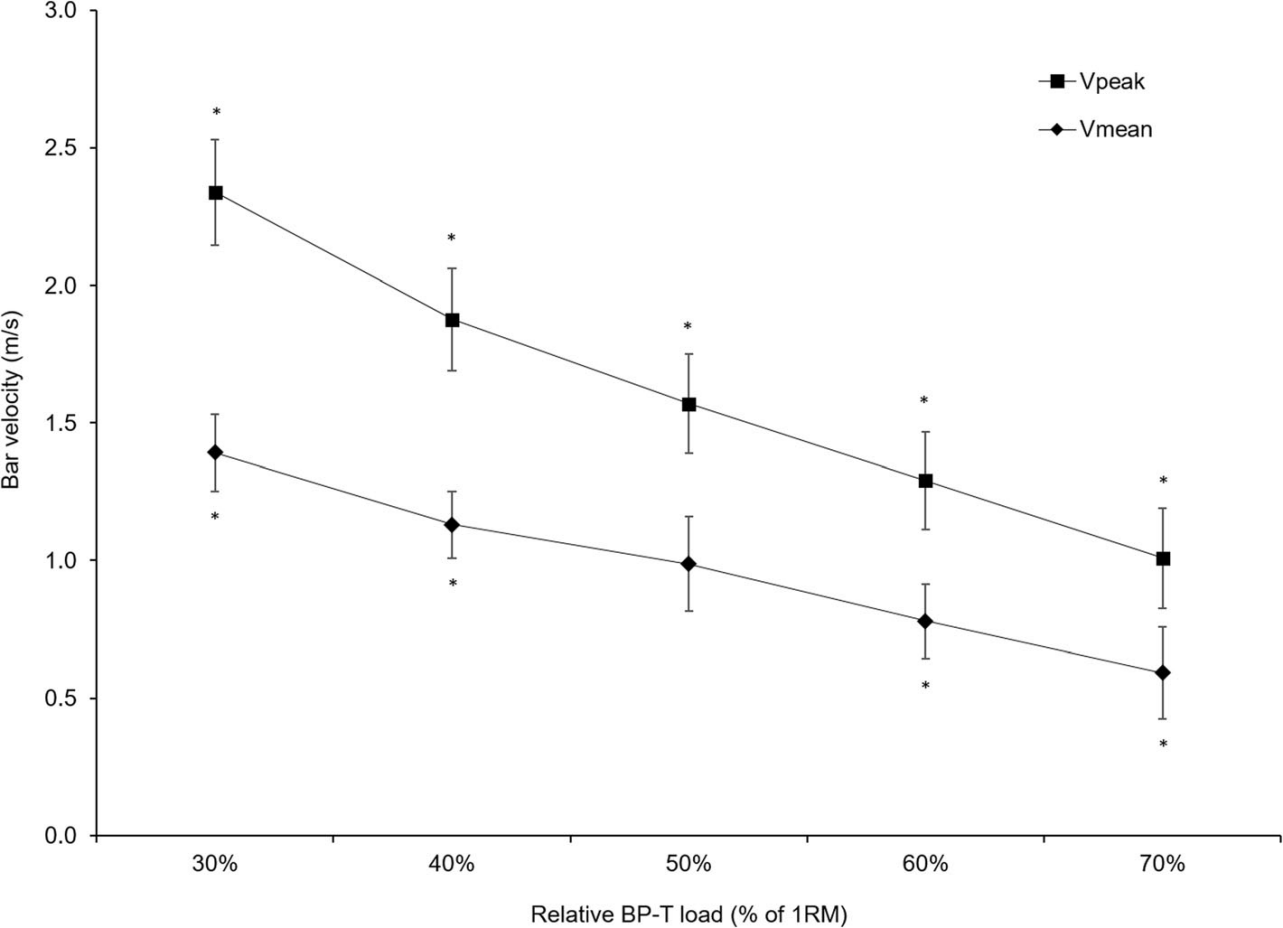
Mean (1SD) mean (V_mean_) and peak bar (V_peak_) vertical velocities at each of the relative loads. * Indicates data significantly different (*P* < 0.01) than the other loads

ANOVA testing indicated that V_mean_ and V_peak_ both showed *large*, significant reductions (*P* < 0.001, *ES* > 1.2) for each respective increase in relative BP-T load, except for V_mean_ between 40 and 50% of 1RM (Fig. 2). Results also showed that the mean values for slope and V_mean_0 at each of the load ranges differed significantly depending on whether V_mean_ or V_peak_ were used in the prediction models (Table 1). Conversely, Load0 data did not differ significantly between either bar velocity measures at each load range. The CV% values range from 7.2 up to 27.5% (Table 2), with the ICC (Table 3) data show excellent reliability for V_peak_ at the lightest range weight whereas only moderate reliability was observed for the weights between 40 and 60%. All other cases showed good reliability. Typically, greater levels of acceptable reliability [32] were recorded for V_peak_ compared to V_mean_.

**Table 1:** Mean (±1SD) values of the slope, abscissa (Load0) and ordinate (V_mean_0) intercept data for each regression line developed using both V_mean_ and V_peak_ across the three loading ranges

Variable	Percent of 1RM		
	30–50%	40–60%	50–70%
Slope using V_mean_	−2.02 (0.52)^a^	−1.76 (0.31)^a^	−1.97 (0.46)^a^
Slope using V_peak_	−3.85 (0.42)	−2.93 (0.71)	−2.81 (0.63)
Load0 using V_mean_ (% of 1RM)	91.9% (15.3)	99.6% (14.9)	103.1% (14.0)
Load0 using V_peak_ (% of 1RM)	89.6% (7.7)^b^	107.8% (23.8)	106.6% (10.2)
V_mean_0 using V_mean_ (m/s)	1.98 (0.22)^a^	1.84 (0.15)^a^	1.97 (0.31)^a^
V_mean_0 using V_peak_ (m/s)	3.47 (0.25)	3.05 (0.41)^a^	2.97 (0.41)^a^

^a^Indicates values differs significantly (*P* < 0.01) from V_peak_ at that load range

^b^Indicates values differ significantly from the actual 1RM at that load range

**Table 2:** CV% values of the slope, abscissa (Load0) and ordinate (V_mean_0) intercept data for each the regression lines developed using both V_mean_ and V_peak_ across the three loading ranges

Variable	Percent of 1RM		
	30–50%	40–60%	50–70%
Slope using V_mean_	25.7	17.6	23.4
Slope using V_peak_	10.9	24.2	22.4
Load0 using V_mean_ (% of 1RM)	16.6	15.0	13.6
Load0 using V_peak_ (% of 1RM)	8.6	22.1	9.6
V_mean_0 using V_mean_ (m/s)	11.1	8.2	15.7
V_mean_0 using V_peak_ (m/s)	7.2	13.4	13.8

**Table 3:** ICC measured versus predicted 1RM

Variable	Percent of 1RM		
	30–50%	40–60%	50–70%
V_mean_	0.868 (0.558–0.966)	0.855 (0.521–0.962)	0.849 (0.506–0.960)
V_peak_	0.967 (0.890–0.990)	0.680 (0.204–0.896)	0.867 (0.604–0.960)

**Fig. 3:**
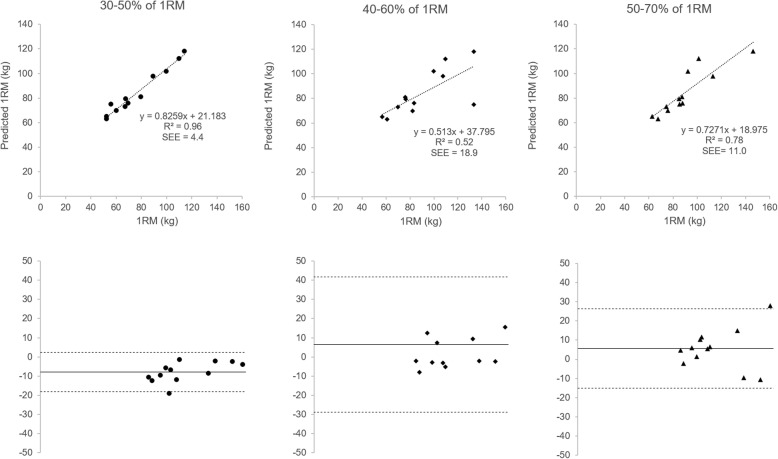
The top row represents the three models to predict 1RM Smith Machine bench press based on mean vertical lifting velocity (V_mean_). The left models are for the loads representing 30–50% of 1RM (●), the middle models for loads 40–60% of 1RM (♦) and the right models for loads representing 50–70% of 1RM (▲). The second row represents the respective Bland-Altman plots for each loading group

**Fig. 4:**
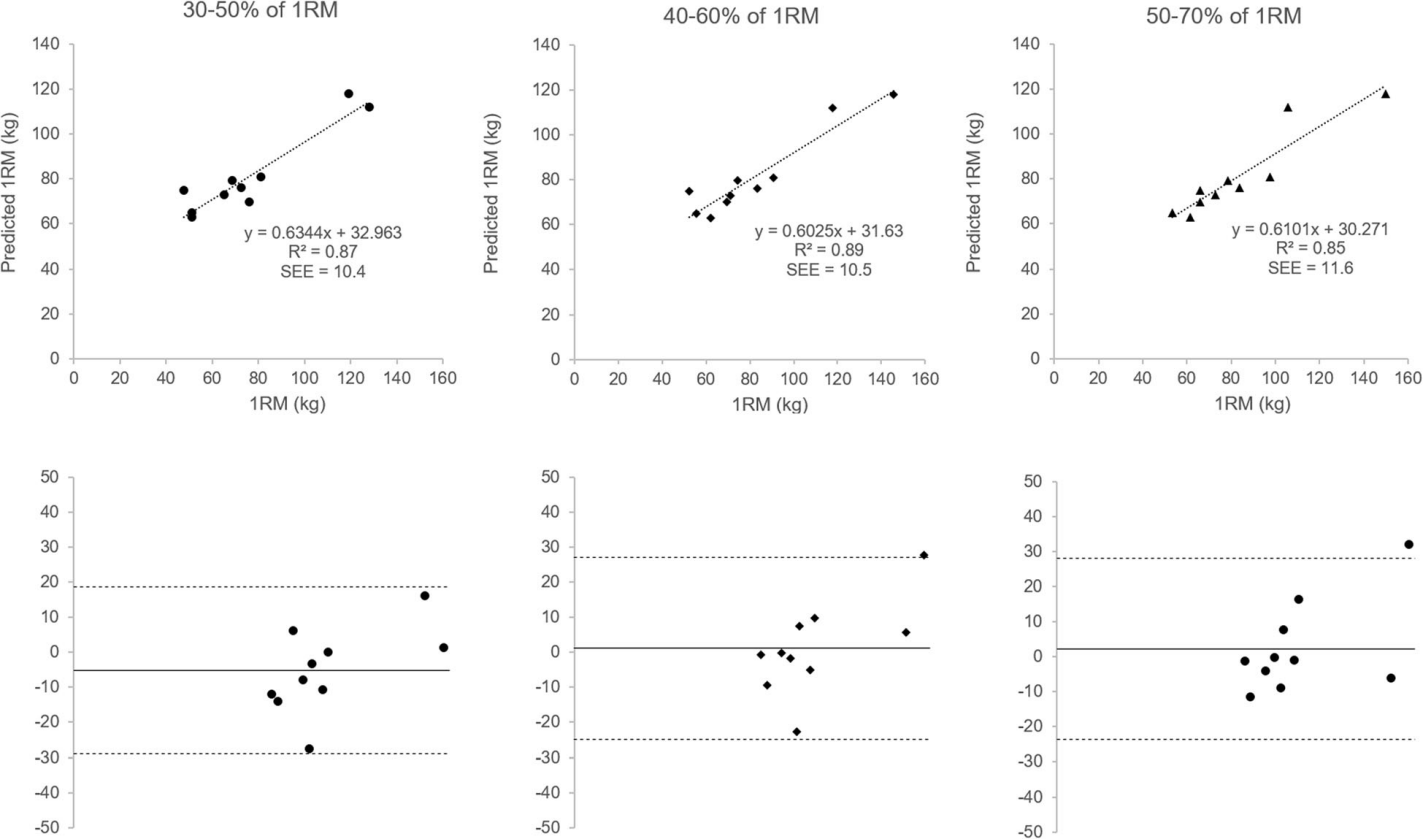
The top row represents the three models to predict 1RM Smith Machine bench press based on peak vertical lifting velocity (V_peak_). The left models are for the loads representing 30–50% of 1RM (●), the middle models for loads 40–60% of 1RM (♦) and the right models for loads representing 50–70% of 1RM (▲). The second row represents the respective Bland-Altman plots for each loading group

There were no noticeable differences in any of the models that used V_mean_ to predict 1RM Smith Machine bench press strength (*R*^*2*^ between 0.85–0.89). Similarly, there were no significant differences between predicted and actual 1RM Smith Machine (*P* = 0.21 to 0.95) when using V_mean_, although the corresponding Bland-Altman plots highlighting some issues with the accuracy of these data (Fig. 3). Conversely, there were significant differences between the predicted and actual 1RM Smith Machine bench press values when using V_peak_ at the lightest of the load ranges to (*P* < 0.001). However, the predicted 1RM values for V_peak_ for these light relative loads (30, 40 and 50% of 1RM) resulted in the most accurate prediction of 1RM bench press strength (Fig. 4), although there was a constant fixed bias towards under estimating 1RM by approximately 9 kg.

## Discussion

This study used the well-established linear-regression techniques proposed by Jidovtseff and coworkers [16] to examined which combination of three relative submaximal loads during BP-T testing results in the best prediction of 1RM Smith Machine bench press strength. We also examined whether regression models developed using V_peak_, rather than the variable suggested by these researchers (V_mean_), provide the best prediction of bench press 1RM. Finally, we examined whether performing a BP-T (instead of an explosive bench press) influences the nature of the regression model when using either of these bar velocity measures.

This study builds upon the findings of Gracia-Ramos and co-workers [24, 25], highlighting that 1RM bench press strength on a Smith Machine can be determined with acceptable levels of precision by examining V_mean_ and V_peak_ during Smith Machine BP-T over three submaximal loads. Perhaps even more importantly, our data suggests that the best and most reliable prediction model was based on relative loads representing just 30, 40 and 50% of 1RM. Importantly, as the prediction is robust for the light relative loads, a rough estimate of the 1RM appears to be sufficient for this method. Although a fixed bias exists to under predict 1RM by approximately 9 kg with using these loads, the accuracy of the model to predict Smith Machine bench press 1RM when using V_peak_ during BP-T is quite high. Additionally, the high precision of this regression model is at least comparable to other established prediction procedures that use more time-consuming protocols and/or also possibly have a greater potential for injury or soreness [5, 7–9, 12–15]. From a practical perspective, our findings suggest that there is no need to test over heavy relative and absolute loads [5, 6] when using the force-load technique to estimate Smith Machine 1RM in recreational and novice level weight trainers [5, 7, 8, 12, 14].

The finding that bar velocity data decreases with increasing relative load is not unique and simply confirms the standard exponential force velocity profile first developed by Hill [33] nearly 80 years ago. Our data for V_mean_ does however contain an anomaly at 50% of 1RM (Fig. 2), suggesting that the V_mean_ may be too gross a measure to be able to detect known changes in performance that occur across our load ranges. Similarly, our results also suggest that V_peak_ is a more effective measure than V_mean_ when using this technique to predict 1RM in regular (but non-athletic) weight trainers with a mean 1RM Smith Machine bench press approximately equivalent to 1 body weight. While these findings are agreement with earlier research [20, 25] other studies favour V_mean_ [24, 26], highlighting that the specific loading regime influences this outcome.

Our V_peak_ data are similar to data from physically active collegiate men performing a similar BP-T task [21], with values between studies differing by less than 0.07 m/s at similar loads. Conversely the slope and V_mean_0 data from our models using V_mean_ to predict 1RM differ considerably from values from the original research using this method [16]. No doubt this is a function of the protocols adopted by these researchers that prevented the participants from releasing the bar (hence performing a dynamic bench press and not a BP-T per se). The use of this approach by these researchers is somewhat surprising as their V_mean_ data would have been effected by a pronounced bar deceleration near the end of the movement [23] and so the accuracy of these data could be optimised. Importantly, V_peak_ during BP-T testing is not only reliable (CV% values between 1.7 and 3.3), but also presents with smaller CV% values than for dynamic bench press movements [21, 25]. From a practical stand point V_peak_ is relatively simple to quantify, as it can be measured using inexpensive devices (e.g. optical encoders or linear position transducers), or estimated using bar throw height. These approaches can be adopted easily in health clubs or commercial gymnasiums and provide acceptable predictions of 1RM that can be used in the development of more effective training programs.

We acknowledge that our testing was based on a relatively small sample of a diverse but specific population of beginning weight training adults, however these samples sizes are relatively common in this domain. Additionally, our sample characteristics are typical for many healthy individuals who attend health clubs and/or commercial strength training facilities. We also acknowledge that it the accuracy of regression models that attempt to predict values outside of the range of the collected data is severely compromised. However, this process is fundamental to all research in this domain and so largely unavoidable. Importantly, we have not suggested that the protocols presented in this project offer an exact estimate of a participant’s Smith Machine 1RM bench press.

## Conclusions

Our results suggest that within this target population reliable estimates of Smith Machine 1RM bench press strength can be achieved using the load-velocity approach with BP-T loads between 30 and 50% of 1RM. We do however acknowledge that the reliability and accuracy of the velocity based method presented here can suffer from fatigue or lack of motivation of the athletes. However, issues such as these are systemic in nearly all strength assessment protocols and can be managed with appropriate testing regimens. We also acknowledge that these data are specific to Smith Machine bench press and BP-T, and may not be transferable to conventional free weight testing. Future research should attempt to confirm these results with a larger sample of participants and conduct appropriate between session reliability assessments. Additionally, it would also be appropriate prospectively compare the incidence of soreness and injury between the methods proposed in this study and traditional 1RM determination.

### Practical applications

Conventional 1RM bench press strength testing exposes people to very high relative loads. Our findings indicate that in normal healthy populations bar velocity data recorded during relatively light load Smith Machine BP-T testing can be used to accurately predict 1RM Smith Machine bench press strength. The large range in the submaximal load range allows practitioners to estimate the 1RM as start point by using a team average, last season values or a weight dependent 1RM to define the submaximal test weights. Additionally, it is simple to determine V_mean_ and V_peak_ during BP-T testing and the linear regression models are easy to apply. Importantly, our results show that the most accurate and reliable models are created from BP-T V_peak_ data (not V_mean_), a variable that developed with minimal post-testing processing, from loads representing just 30, 40 and 50% of 1RM. Using the approach described in our study exercise programmers can predict 1RM Smith Machine bench press strength and monitor performance enhancement with acceptable accuracy without the need to expose their clients to extremely heavy loads, or lift to fatigue protocols.

## Abbreviations

1RM: One repetition maximum
BM: Body mass
BP-T: Bench press throw
CV%: Coefficient of variance
ICC, 3,1: Intra class correlations
Load0: The abscissa from the linear regression equation derived from load and mean vertical propulsive lifting velocity
LPT: Linear position transducer
Slope: The gradient of the linear regression equation derived from load and mean vertical propulsive lifting velocity
V_mean_: Mean vertical propulsive velocity
V_mean_0: The ordinate from the linear regression equation derived from load and mean vertical propulsive lifting velocity
V_peak_: Peak vertical lifting velocity
